# Public and occupational health risks related to lead exposure updated according to present-day blood lead levels

**DOI:** 10.1038/s41440-022-01069-x

**Published:** 2022-10-18

**Authors:** Yu-Ling Yu, Wen-Yi Yang, Azusa Hara, Kei Asayama, Harry A. Roels, Tim S. Nawrot, Jan A. Staessen

**Affiliations:** 1 Research Unit Environment and Health, KU Leuven Department of Public Health and Primary Care, University of Leuven, Leuven, Belgium; 2 Department of Cardiology, Shanghai General Hospital, Shanghai Jiao Tong University School of Medicine, Shanghai, China; 3 Division of Drug Development and Regulatory Science, Faculty of Pharmacy, Keio University, Tokyo, Japan; 4 Department of Hygiene and Public Health, Teikyo University School of Medicine, Tokyo, Japan; 5 Research Unit Hypertension and Cardiovascular Epidemiology, KU Leuven Department of Cardiovascular Sciences, University of Leuven, Leuven, Belgium; 6 Non-Profit Research Association Alliance for the Promotion of Preventive Medicine, Mechelen, Belgium; 7 Center for Environmental Sciences, Hasselt University, Diepenbeek, Belgium; 8 Biomedical Science Group, Faculty of Medicine, University of Leuven, Leuven, Belgium

**Keywords:** Environmental medicine, Hypertension, Lead, Mortality, Occupational medicine

## Abstract

Lead is an environmental hazard that should be addressed worldwide. Over time, human lead exposure in the western world has decreased drastically to levels comparable to those among humans living in the preindustrial era, who were mainly exposed to natural sources of lead. To re-evaluate the potential health risks associated with present-day lead exposure, a two-pronged approach was applied. First, recently published population metrics describing the adverse health effects associated with lead exposure at the population level were critically assessed. Next, the key results of the Study for Promotion of Health in Recycling Lead (SPHERL; NCT02243904) were summarized and put in perspective with those of the published population metrics. To our knowledge, SPHERL is the first prospective study that accounted for interindividual variability between people with respect to their vulnerability to the toxic effects of lead exposure by assessing the participants’ health status before and after occupational lead exposure. The overall conclusion of this comprehensive review is that mainstream ideas about the public and occupational health risks related to lead exposure urgently need to be updated because a large portion of the available literature became obsolete given the sharp decrease in exposure levels over the past 40 years.

## Graphical abstract

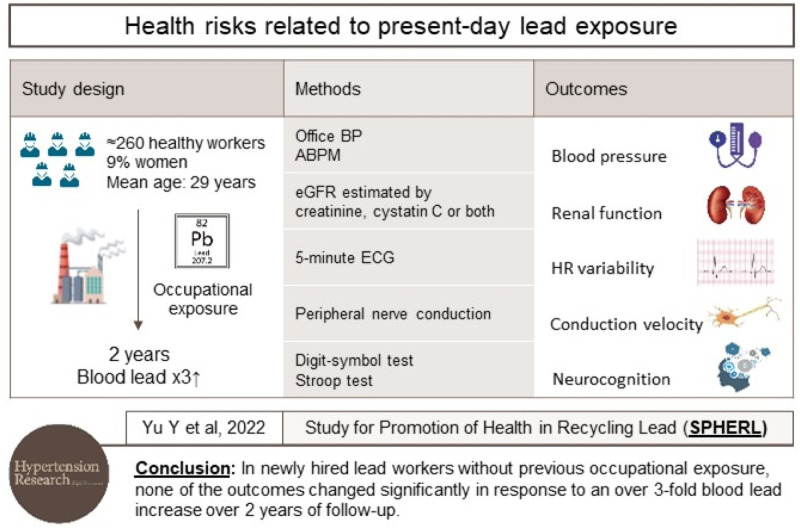

## Introduction

Lead is an environmental toxicant. High levels of lead exposure can lead to hypertension and renal failure, as observed in the past in occupational settings or in the general population (e.g., by the consumption of moonshine whiskey) [1, 2]. However, the National Health Examination Survey (NHANES) demonstrated that mean blood lead levels among American adults decreased from 13.1 µg/dl in NHANES II (1976–1980) [3] to 2.76 µg/dL in NHANES III (1988–1994) [3] and further to 1.64 µg/dl in NHANES IV (1999–2002) [4, 5]. Over time, increasingly strict environmental regulations led to the banning of lead-containing paint (1976) [6], the phasing out of leaded gasoline (1995) [6], the elimination of lead as a construction material, the replacement of lead pipes in drinking water distribution, the elimination of lead solder in food cans and the compulsory and systematic recycling of lead batteries and other lead waste. In developed nations, the average blood lead concentration in the general population currently approaches 1.5 μg/dl, which is close to the estimated blood lead concentration in preindustrial humans (2 μg/dl), only exposed to natural sources, as estimated by the Global Burden of Disease (GBD) Consortium [7]. However, linear regression analysis of blood lead on bone lead obtained pairwise from environmentally or occupationally exposed humans [8, 9] revealed that the preindustrial blood lead concentration in humans might have been as low as 0.016 μg/dl. It is unclear how blood and bone samples collected in the early 1990s and extrapolation justify this extremely low blood lead level for individuals only exposed to lead from natural sources.

Our studies in the field of environmental medicine span four decades but did not provide convincing evidence supporting environmental lead exposure as being causally related to hypertension [10–12], renal dysfunction [13–15] or cardiovascular disease [16, 17]. Given this research track, the aims of the present review were to identify sources of bias in recent publications [18] examining the association between adverse health outcomes and lead exposure and to summarize the key results of the Study for Promotion of Health in Recycling Lead (SPHERL; NCT02243904) [19]. To our knowledge, SPHERL is the first prospective study that accounted for interindividual variability between people in their vulnerability to the toxic effects of human lead exposure by assessing participants’ health status before and after lead exposure [19], an issue identified as a research priority in a meta-analysis published in 2002 [20]. As an introduction to the field, the toxicokinetics of lead in humans are first summarized.

## Toxicokinetics of lead in humans

Lead enters the body primarily through inhalation and ingestion. Currently, adults are mainly exposed by breathing in lead-contaminated fine particulate and fumes at work or during leisure time activities that involve lead. Exposure of the general population to ambient air is generally due to respirable particles capable of deep lung penetration and deposition [21]. Once the finest dust particles reach the lung alveoli, they readily pass the air‒blood barrier and are subsequently system-wide distributed via the bloodstream. Occupational exposure entails coarser aerosols that deposit in the upper airways and then translocate to the gastrointestinal tract by mucociliary clearance, where gastrointestinal uptake kinetics prevail (5-10% uptake). The lead in air to lead in blood slope is approximately 2 for ambient exposure and 0.05 for occupational exposure [21].

Lead is a cumulative toxicant, 90–95% of which is stored in bone, from which it is recirculated with a half-life of 20–25 years [22, 23]. Blood lead, 99% of which is carried by red blood cells, reflects recent exposure over the past 1–2 months and the amount of lead released and recirculated from bone stores [22]. Both bone [23, 24] and blood [13, 23, 24] lead increase with advancing age. Bone lead levels are correlated with blood lead [23, 24] and explain approximately 20% of the variance in blood lead, depending on seasonality [23] and hormonal and other endogenous and environmental stimuli influencing the balance between bone formation and resorption [24]. Recirculation of lead from bone explains why there is a lag time for blood lead to decline when environmental [11] or occupational [22] lead exposure decreases.

## Sources of bias in the literature

Relevant publications are the NHANES III results and the articles published by the GBD consortium.

### Mortality in relation to blood lead in NHANES III

The cross-sectional NHANES III survey (1988–1994) involved the collection of clinical variables, questionnaire data and biochemical measurements, including blood lead, among a representative sample of the adult population of the United States [25–27]. Blood lead was measured by graphite furnace atomic absorption spectrophotometry. The detection limit was 1.0 µg/dl. For participants (8%) with blood lead levels below the detection limit, a level of 0.7 µg/dl was imputed [25–27]. These NHANES III baseline data were linked with the National Death Index, and probabilistic matching was used based on 12 identifiers for each participant to ascertain vital status and the cause of death. The follow-up period was the time between the baseline examination date and the date of death or the participant’s 90th birthday, whichever came first. In the most recent NHANES III report, which will be discussed in detail, the censoring date was 31 December 2011 [27].

In 14,289 individuals (47.9% men, Table 1 [27]), the multivariable-adjusted hazard ratios expressing the risk of an increase in blood lead from the 10th to the 90th percentile (1.0–6.7 µg/dl) were 1.37 (95% confidence interval [CI], 1.17–1.60), 1.70 (CI, 1.30–2.22), and 2.08 (CI, 1.52–2.85) for total, cardiovascular and coronary mortality, respectively. From individual measures of blood lead and their associated hazard ratios, the population attributable fraction (PAF [28, 29]), i.e., the adverse health outcomes attributable to lead exposure, was then computed as the integral of the hazard ratios at each blood lead level weighted by the logarithmically transformed population distribution of blood leads over the total range from 0.70 to 56.0 µg/dl. The PAFs amounted to 18.0% (CI, 10.9–26.1%) for total mortality, 28.7% (CI, 15.5–39.5%) for cardiovascular mortality, and 37.4% (CI, 23.4–48.6%) for coronary mortality. Given the annual overall mortality (n = 2,288,888), cardiovascular mortality (*n* = 891,896), and coronary mortality (*n* = 494,652) in the United States and assuming that blood lead concentrations might be reduced to 1.0 µg/dl or less, the number of preventable deaths amounted to 412,000 (CI, 250,000–598,000) for total mortality, 256,000 (CI, 138,000–352,000) for cardiovascular mortality, and 185,000 (CI, 116,000–241,000) for coronary mortality.

**Table 1:** Mortality in 14,289 NHANES III participants followed up until 31 December 2011

Variable	All participants	Results by thirds of the blood lead distribution			*p*
Blood lead range (µg/dl)	0.7-56.0	<2.0	2.0-3.7	≥3.8	
Risk factors					
Black, %	10.2	9.1	9.2	12.1	0.0004
Men, %	47.9	24.6	49.2	68.3	<0.0001
Age, years	44.1	37.8	44.8	48.2	<0.0001
Body mass index					
<25 kg/m^2^, %	44.6	49.4	42.8	42.0	<0.0002
25–29.9 kg/m^2^, %	33.0	27.0	24.5	36.9	<0.0001
≥30 kg/m^2^, %	22.4	23.6	22.7	21.1	0.13
Current smoking, %	34.9	23.0	33.0	47.8	<0.0001
Alcohol consumption, %					
<4 per month, %	63.2	73.3	62.3	54.8	<0.0001
≥4 per month, %	36.8	26.7	37.7	45.2	<0.0001
Hypertension, %	17.5	9.6	18.0	24.3	<0.0001
<$20 000 annual income, %	31.9	27.7	24.0	37.4	<0.0001
Total mortality					
Deaths, *n* (%)	4422 (30.9)	631 (13.2)	1340 (28.1)	2451 (51.5)	
Hazard ratios (95% CI)					
Primary analysis	1.37 (1.17–1.60)	…	…	…	…
Sensitivity analyses					
Blood lead <5 µg/dl	1.38 (1.15–1.66)	…	…	…	…
HT + treatment status	1.38 (1.18–1.61)	…	…	…	…
SBP + DBP (continuous)	1.36 (1.16–1.58)	…	…	…	…
Cardiovascular mortality					
Deaths, n (%)	1801 (12.6)	218 (4.6)	552 (11.6)	1031 (21.6)	
Hazard ratios (95% CI)					
Primary analysis	1.70 (1.30–2.22)	…	…	…	…
Sensitivity analyses					
Blood lead <5 µg/dl	1.95 (1.46–2.60)	…	…	…	…
HT + treatment status	1.73 (1.32–2.27)	…	…	…	…
SBP + DBP (continuous)	1.68 (1.28–2.19)	…	…	…	…
Coronary mortality					
Deaths, *n* (%)	988 (6.9)	112 (2.4)	284 (6.0)	592 (12.4)	
Hazard ratio (95% CI)					
Primary analysis	2.08 (1.52–2.85)	…	…	…	…
Sensitivity analyses					
Blood lead <5 µg/dl	2.57 (1.56–4.52)	…	…	…	…
HT + treatment status	2.13 (1.55–2.93)	…	…	…	…
SBP + DBP (continuous)	2.07 (1.55–2.84)	…	…	…	

HT, SBP, DBP indicate hypertension, systolic blood pressure, diastolic blood pressure, respectively. Data were extracted from ref. [27]. Of 18,825 participants enrolled, 1795 had no medical examination or home visit, 1419 were excluded because of missing blood lead or urinary cadmium, 1314 because of missing covariables, and 8 because of missing identifiers to match with the national registry, leaving 14,289 for statistical analysis. Hazard ratios, given with 95% confidence interval, represent the relative risk for an increase in blood lead from 1.0 to 6.7 µg/dl (10th–90th percentile interval). Hazard ratios accounted for ethnicity (White, Black, or Mexican-American), sex, the linear and squared terms of age, body mass index (categorical), hypertension (blood pressure ≥140 mmHg or ≥90 mmHg diastolic), smoking status (never, current, or former), alcohol consumption (<4 vs ≥ 4 drinks per month), serum cholesterol, glycated hemoglobin, urinary cadmium (categorized), physical activity (categorized into none, 1-14 and ≥15 times in the previous month), annual income (<vs ≥ $20,000), and the healthy eating index (categorized). Sensitivity analyses were conducted by including only participants with blood lead <5 µg/dl (relative risk given for the 10th-80th percentile interval), considering treatment status in the definition of hypertension, and entering systolic and diastolic blood pressure as continuous covariables in the models to replace hypertension (categorical). To convert blood lead concentration from µg/dl to µmol/L, multiply by 0.0483. An ellipsis indicates that in ref. [27] hazard ratios were not given for increasing categories of blood lead. Reproduced from ref. [27], which was published was an open-access article under the terms of the Creative Commons Attribution Non-Commercial-NoDerivs License

This 2018 NHANES report (Table 1 [27]), based on historical blood lead (1988–1994), has little relevance for public health policies in the third decade of the twenty-first century for the following reasons. First, the blood lead levels recorded in NHANES III are not representative of current lead exposure. To a large extent, these levels reflected the recirculation of lead from earlier bone stores, which in many participants accrued from the first decades of the twentieth century onward, when lead was still highly prevalent in the environment in the United States. In our analyses of 12,725 NHANES IV participants examined from 2003 to 2010 [5], the geometric mean blood concentration in all participants was 1.41 µg/dl, with lower levels in women than men (1.25 vs. 1.80 µg/dl) and in Whites than in Blacks and Hispanics (1.46 vs. 1.57 µg/dl). All blood lead levels were below 30 µg/dl [5]. Second, PAF was calculated as the proportional decline in mortality that would occur if the blood lead concentrations of all participants were reduced to a reference level of 1.0 µg/dl or less [27], which is an unfeasible target, given lead exposure from natural sources and food. This very low null-effect blood lead concentration substantially inflated the hazard ratios and PAFs associated with blood lead. Third, hypertension as the causal pathway linking mortality to environmental or occupational lead exposure is a deeply rooted paradigm, based on research dating back more than half a century ago [30, 31]. The NHANES III report itself [27] argued against this mechanistic pathway, given that models accounting for hypertension and hypertension treatment or adjusted for systolic and diastolic blood pressure as continuously distributed variables barely affected the hazard ratios (Table 1). Along similar lines, in a meta-analysis of summary statistics extracted from 31 studies involving 58,518 participants, all published before February 2001 [32], doubling of blood lead was only associated with a marginally higher blood pressure. The pooled estimates averaged 1.0 mmHg (CI, 0.5–1.4 mmHg) systolic and 0.6 mmHg (CI, 0.4–0.8 mmHg) diastolic. Furthermore, in a prospective population study of 728 individuals (50.7% women; age range, 20–82 years), blood pressure was measured conventionally at baseline (1985–1989) and at follow-up (1991–1995) and by 24-h ambulatory monitoring at follow-up [11]. Over a median follow-up of 5.2 years (range, 3.5–8.4 years), the geometric mean blood lead concentration dropped by 32% from the baseline level of 8.7 µg/dl (range, 1.7-72.5 µg/dl). The small changes in the systolic/diastolic blood pressure on conventional measurement (−1.5/+1.7 mmHg) were unrelated to the blood lead concentration at baseline or to the changes in this exposure biomarker over follow-up. Similarly, the 24-h ambulatory blood pressure was not associated with blood lead at baseline or follow-up [11]. A recent NHANES report with data from 1999–2016 [33] included 30,467 participants aged 20–79 years. Non-Hispanic Black men (*n* = 3006) had the highest mean blood lead level (2.20 μg/dl), compared with 3814 Hispanic men (2.18 μg/dl) and 6989 non-Hispanic White men (1.89 μg/dl). A similar ethnic gradient in blood lead was observed among women: 1.49 μg/dl in 3256 non-Hispanic Black women, 1.30 μg/dl in 4130 Hispanic women, and 1.30 μg/dl in 7078 non-Hispanic White women. In multivariable-adjusted logistic regression models [33], hypertension was not associated with blood lead (odds ratio, 1.002; CI, 0.983–1.021).

The number of deaths in the top third of the NHANES III blood lead distribution amounted to 2451 (55.4% of all-cause mortality; Table 1) [27]. The 2011 National Vital Statistics Report [34] listed cause-specific mortality corresponding in time with the end of the 20-year follow-up of the NHANES III participants [27]. Standardized per 100,000 deaths, from 45 to 84 years, malignancies contributed 434 more deaths to all-cause mortality than cardiovascular disease, whereas only from age 85 onward did heart disease overtake malignant disease, contributing 2435 extra deaths. The NHANES III models [27] were unadjusted for the competing risks of fatal cardiovascular and noncardiovascular diseases, both contributing to all-cause mortality [35, 36]. Finally, a major limitation of the NHANES III studies [25–27] was their focus on mortality. The introduction of stroke units and the wide availability of invasive coronary care, thrombolysis and percutaneous vascular interventions have reduced the case-fatality rate of most cardiovascular complications of hypertension. Therefore, not accounting for nonfatal events limits the generalizability of the NHANES III reports [25–27].

### Global burden of disease reports

A disability-adjusted life year (DALY) is a summary metric that reflects the sum of years lived with a disability and the years of life lost. This metric therefore reflects both quality of life and premature mortality [37]. The GBD consortium proposed that from the age of 25 years onward, there is a causal association between systolic blood pressure and lead exposure [38, 39] Mediated via blood pressure, lead exposure was unrealistically assumed to cause a wide range of cardiovascular diseases, including right heart disease; ischemic heart disease; ischemic, hemorrhagic and other nonischemic stroke; hypertensive heart disease; aortic aneurysm; the aggregate of cardiomyopathy, myocarditis and endocarditis; the aggregate of atrial fibrillation and flutter; pulmonary vascular disease; other cardiovascular disease; and chronic kidney disease [40]. If evidence was only available for the relative risk of either morbidity or mortality, the assumption was that estimates of relative risk would equally apply to both fatal and nonfatal outcomes. In 2010, high blood pressure was the leading single risk factor globally, accounting for 9.4 million deaths (95% uncertainty interval [UI], 8.6–10.1 million) and 7.0% (UI, 6.2–7.7%) of global DALYs [37]. For environmental lead exposure, these estimates were 0.67 million deaths (UI, 0.58-0.78 million) and 0.56% of DALYs lost (UI, 0.47–0.66%) [37]. Worldwide, for both sexes and all ages combined, high blood pressure moved up in the global risk factor ranks from rank 4 in 1990 to rank 1 (UI, 1–2) in 2010 and environmental lead exposure from rank 30 to rank 25 (UI, 23–29) [37].

The GBD investigators listed among the possible limitations of their results (i) residual confounding; (ii) uncertainty as to the extent to which effect sizes were generalizable; and (iii) the impossibility of accounting for temporal changes in the exposure to risk factors. Thus, the GBD statistics fell short in accounting for the steady global decline in environmental lead exposure. This might explain why globally, despite declining environmental exposure [3–5, 11], environmental lead exposure increased from risk factor rank 30 in 1990 to rank 25 in 2010 [37]. Furthermore, the issue of residual confounding requires calculating PAF for clusters of risk factors, rather than for a single risk indicator. Indeed, cardiovascular risk factors [41–43] and exposures to various environmental pollutants [10, 14, 44] cluster within individuals, such as poverty, unhealthy lifestyle habits, poor housing conditions, and lead exposure in the NHANES surveys. The GBD estimates did not account for coexposures to risk factors and environmental pollutants. According to WHO demographic data, in 2010, the population of the United States (309 million) represented approximately 4.5% of the world’s population (6.9 billion). Interestingly, if the statistics of the GBD 2012 report are truly generalizable (PAF, 0.67 deaths worldwide [37]), preventable deaths related to environmental lead exposure in the United States would amount to approximately 30,150 per year, an estimate more than 10-fold smaller than that proposed in the NHANES III report [27].

## SPHERL

### Design of SPHERL

SPHERL is a longitudinal study of newly hired lead workers without known previous occupational exposure. The participants were employed at battery manufacturing and lead recycling plants in the United States. SPHERL complied with the Helsinki Declaration for investigations in humans. The Ethics Committee of the University Hospitals Leuven (Belgium) approved the study protocol (No B322201421631), which has been published in detail [19]. The health of the labor force enrolled in the SPHERL cohort was protected in compliance with the US Occupational Safety and Health Administration Standard (www.osha.gov/laws-regs/regulations/standardnumber/1910/1910.1025). This standard includes regular medical monitoring, proper workplace ventilation, and the obligatory use of personal protective equipment. The coprimary endpoints for SPHERL were the changes in blood pressure and renal function [19]. The secondary endpoints included the autonomous nervous regulation of the cardiovascular system, as captured by heart rate variability (HRV), neurocognitive function and peripheral nerve conductivity.

The workers underwent follow-up visits 1 and 2 years after enrollment. Detailed diagrams describing the flow of participants and the number of workers excluded from the statistical analyses have been published for each endpoint [45–48]. In medical facilities separate from the production sites, the study nurses obtained venous blood samples after participants had fasted for 8 h. The materials used for blood collection, including test tubes, needles and caps, were certified as lead free (Becton, Dickinson and Company, Franklin Lakes, NJ). The nurses thoroughly cleansed the brachial venipuncture site and kept the tubes for the measurement of blood lead closed. Blood lead was determined by inductively coupled plasma‒mass spectrometry at a single laboratory certified for blood lead analysis in compliance with the provisions of the Occupational and Health Administration Lead Standard, 29CFR 1910.1025 (Occupational Safety and Health Administration). The laboratory participated in the US CDC Blood Lead Proficiency Testing Program. Before analysis, specimens were digested in nitric acid and spiked with an iridium internal standard. The blood lead detection limit was 0.5 µg/dl. The deviation from known lead standards analyzed along with the samples in each test run was less than 10%.

### Changes in blood lead

In the most recently published SPHERL article focusing on renal function [48], the geometric mean blood lead concentration was 4.22 μg/dl (interquartile range [IQR], 2.50–8.30 μg/dl) at baseline, 14.1 μg/dl (IQR, 9.95–22.2 μg/dl) at the first follow-up visit, and 14.1 μg/dl (IQR, 11.0–23.1 μg/dl) at the last follow-up. The last-follow-up-to-baseline blood lead concentration ratio averaged 3.34 (CI, 2.98–3.76; *P* < 0.001; Fig. S1). Changes in the blood lead concentration were similar in the SPHERL publications addressing the other study endpoints [45–47].

### Blood pressure and hypertension

At the study sites, trained nurses applied current guidelines to measure office blood pressure at the brachial artery [49]. After the workers had rested for 5 min in the sitting position, the nurses obtained five consecutive blood pressure readings to the nearest 2 mmHg by auscultation of the Korotkoff sounds using standard mercury sphygmomanometers. For analysis, the five readings were averaged. The ambulatory blood pressure was recorded on the same arm as the office blood pressure with similarly sized cuffs using validated [50] oscillometric Mobil-O-Graph 24-h PWA monitors (I.E.M. GmbH, Stolberg, Germany). The monitors were programmed to obtain readings at 15-min intervals during waking hours and every 30 minutes during sleep. Mean 24-hour blood pressure was the average of the awake and asleep blood pressures weighted for the duration of the awake and asleep periods. Office and ambulatory blood pressure were categorized according to the 2017 American College of Cardiology (ACC)/American Heart Association (AHA) guidelines [49].

**Fig. 1:**
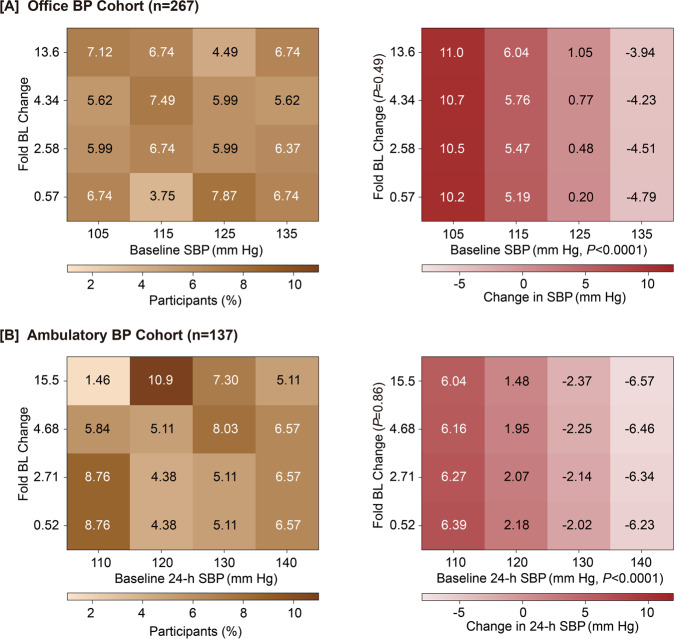
Heatmaps relating the change in office (**A**) and 24-h ambulatory (**B**) systolic blood pressure to the fold change in blood lead from baseline to last follow-up. BL indicates blood lead, and SBP refers to systolic blood pressure. Associations were derived by mixed models including the individual as a random effect. Models were adjusted for ethnicity (white vs. other), sex, age, body mass index at baseline, change in body weight during follow-up, the baseline value of blood lead, and the baseline values of and the changes during follow-up in heart rate, smoking status, total-to-HDL serum cholesterol ratio, γ-glutamyltransferase, and serum creatinine. The percentage of workers contributing to the cross-classification between the baseline blood pressure (horizontal axis) and the fold change in blood lead blood lead is given for each analysis run. Reproduced from ref. [47], which was published was an open-access article under the terms of the Creative Commons Attribution Non-Commercial-NoDerivs License

Office blood pressure was measured in 267 participants (11.6% women, mean age at enrollment, 28.6 years) and the 24-ambulatory in 137 at two follow-up visits. Fully adjusted changes in systolic/diastolic blood pressure associated with a doubling of the blood lead ratio were 0.36/0.28 mmHg (CI, 0.55–1.27/0.48–1.04 mmHg) for office blood pressure and 0.18/0.11 mmHg (2.09–1.74/1.05–1.27 mmHg) for 24-h blood pressure. The adjusted hazard ratios for moving up hypertension categories associated with a doubling of the blood lead concentration were 1.13 (0.93–1.38) and 0.84 (0.57–1.22) for the office and the 24-h blood pressure, respectively. Heatmaps demonstrated, as is true for all clinical measurements [51], that the baseline blood pressure was the main determinant of blood pressure at follow-up (Fig. 1). Due to regression to the mean, workers with low blood pressure at enrollment were more likely to experience an increase in their office and ambulatory blood pressure or to move up across the ACC/AHA hypertension categories, whereas the opposite was the case for workers in the top tail of the baseline blood pressure distribution. However, there was no systematic shift in the blood pressure distributions from baseline to the last follow-up. During the 2-year follow-up, there was not a single case of the wide array of cardiovascular diseases to be associated with lead exposure according to the 2012 GBD report [40].

### Renal function

The glomerular filtration rate was estimated from serum creatinine (eGFRcrt), serum cystatin C (eGFRcys) or both (eGFRcc) as defined in Table S1 [52]). Serum osmolality (mOsm/kg) was computed as 2 × (serum Na+ [mmol/L]) + (blood glucose [mg/dl]/18) + (blood urea nitrogen [mg/dl]/2.8) [53]. In fully adjusted mixed models (Table 2), which also accounted for the within-participant clustering of the 1- and 2-year follow-up data, a 3-fold increment in blood lead was not significantly correlated with changes in eGFR, with estimates amounting to −0.86 (95% CI, −2.39, 0.67), −1.58 (−3.34, 0.18) and −1.32 (−2.66, 0.03) ml/min/1.73 m2 for eGFRcrt, eGFRcys or eGFRcc, respectively [48]. Baseline blood lead did not materially modify these estimates, but the baseline eGFR was a major determinant of eGFR changes showing regression to the mean during follow-up (Fig. S2). Responses of serum osmolarity, urinary gravity or the urinary albumin-to-creatinine ratio were also unrelated to the blood lead increment. The age-related decreases in eGFRcrt, eGFRcys, and eGFRcc were −1.41, −0.96, and −1.10 ml/min/1.73 m^2^, respectively.

**Table 2:** Association between changes in renal function and change in blood lead

Variable	Unadjusted		Adjusted		Fully adjusted	
	*β* (95% CI)	*p*	*β* (95% CI)	*p*	*β* (95% CI)	*p*
Serum creatinine, ×10^-2^ mg/dl	0.28 (−1.37, 1.92)	0.74	0.74 (−0.74, 2.23)	0.32	0.72 (−0.81, 2.26)	0.35
Serum cystatin C, ×10^-2^ mg/L	0.65 (−0.89, 2.19)	0.41	1.05 (−0.42, 2.52)	0.16	1.18 (−0.33, 2.68)	0.12
eGFRcrt, ml/min/1.73 m^2^	−0.43 (−2.08, 1.23)	0.61	−0.86 (−2.34, 0.61)	0.25	−0.86 (−2.39, 0.67)	0.27
eGFRcys, ml/min/1.73 m^2^	−0.88 (−2.67, 0.91)	0.33	−1.42 (−3.13, 0.30)	0.11	−1.58 (−3.34, 0.18)	0.078
eGFRcc, ml/min/1.73 m^2^	−0.75 (−2.21, 0.71)	0.31	−1.21 (−2.51, 0.09)	0.069	−1.32 (−2.66, 0.03)	0.055
Serum osmolality, mOsm/kg	0.08 (−0.57, 0.74)	0.80	0.02 (−0.47, 0.50)	0.95	−0.05 (−0.55, 0.46)	0.85
Serum sodium, mmol/l	0.13 (−0.19, 0.45)	0.42	0.09 (−0.16, 0.34)	0.48	0.06 (−0.20, 0.31)	0.66
Blood glucose, mg/dl	−2.19 (−4.67, 0.29)	0.084	−0.78 (−2.91, 1.34)	0.47	−0.93 (−3.07, 1.22)	0.40
Insulin, %	−4.44 (−15.4, 7.94)	0.46	−4.30 (−14.6, 7.26)	0.45	−3.75 (−13.6, 7.19)	0.48
Blood urea nitrogen, mg/dl	−0.13 (−0.63, 0.38)	0.62	−0.18 (−0.61, 0.25)	0.40	−0.13 (−0.57, 0.32)	0.57
BUN-to-SCRT ratio	−0.23 (−0.76, 0.29)	0.38	−0.40 (−0.83, 0.03)	0.067	−0.35 (−0.80, 0.09)	0.12
Urine specific gravity, ×10^-2^	−0.03 (−0.14, 0.09)	0.65	−0.03 (−0.11, 0.06)	0.54	−0.00 (−0.09, 0.08)	0.94
ACR, %	1.31 (−8.03, 11.6)	0.79	−0.37 (−8.34, 8.29)	0.93	0.23 (−7.94, 9.12)	0.96

eGFRcrt, eGFRcys, and eGFRcc refer to the glomerular filtration rate estimated from serum creatinine, serum cystatin C or both [52]. BUN-to-SCRT ratio is the ratio of blood urea nitrogen (mg/dl) to serum creatinine (mg/dl). Changes in serum insulin and the urinary albumin-to-creatinine ratio are expressed as percentage differences from baseline to follow-up. Association sizes (*β*), given with 95% confidence interval, express the change in the dependent variable for a threefold increase in the blood lead concentration. Adjusted models accounted for sex, age, follow-up duration, the time of day of blood sampling (nighttime vs daytime), and the baseline renal function measure being analyzed. Fully adjusted models additionally accounted for baseline body mass index, change in body weight, and the baseline values of and changes during follow-up in smoking status, mean arterial pressure, antihypertensive medication (yes vs no), the total-to-HDL cholesterol ratio and γ-glutamyltransferase. Reproduced from ref. [48], which was published was an open-access article under the terms of the Creative Commons Attribution Non-Commercial-NoDerivs License

In addition to regression to the mean in the estimates of eGFR, the SPHERL renal function article highlighted the importance of accounting in longitudinal studies for concealed, albeit not unexpected, confounders. Figure S3 shows that eGFRcrt, eGFRcys and eGFRcc were 8.96, 4.68 and 7.18 ml/min/1.73 m^2^ lower, respectively, when determined from night shift blood samples compared with morning or evening blood samples. Both serum creatinine and serum cystatin C, from which eGFR is derived, show a diurnal rhythm with little influence of meals or meat ingestion on serum cystatin C, whereas these confounders increase serum creatinine [54]. Along similar lines, during sleep, urine flow decreases, and the tubular reabsorption of water increases [55, 56]. The newly hired workers recruited into SPHERL transitioned not only from environmental to occupational lead exposure but also from a sedentary to a physically demanding lifestyle. Based on published tables [57], the jobs offered to the workers required an energy expenditure of 6 to more than 8 metabolic equivalents, defined as the amount of oxygen consumed while resting in the sitting position. In young adults, exercise reduces renal plasma flow and eGFR with smaller effects on eGFRcys than on eGFRcrt [58]. Strenuous physical work is also associated with sodium and water loss through sweating and an increased respiration rate and with higher insulin sensitivity, thus leading to increases in serum sodium and urine specific gravity and decreases in blood glucose during follow-up compared with baseline (Table 2). Few previous studies accounted for these confounders.

### Autonomous nervous system function

HRV was measured from 5-min ECG recordings in the supine and standing positions using Cardiax software, V.4.14.0 (International Medical Equipment Development, Budapest, Hungary). The time interval between the supine and standing ECG recordings was not standardized but lasted from 1 to 3 min. Cardiax software allowed the exporting of all ECG measurements into an Excel worksheet, which was subsequently imported into SAS version 9.4, using standardized programming statements, thereby excluding any observer-induced bias. The software computes the power spectrum in the frequency domain by fast Fourier transform and autoregressive modeling and provides the low-frequency (0.04–0.15 Hz) and high-frequency (0.15–0.40 Hz) HRV components in milliseconds and the low-to-high-frequency ratio. The Fourier approach consisted of a mathematical transform that decomposes the heart rate signal changing over time into its constituent frequencies. The autoregressive approach derived HRV by regressing heart rate at a given time as a response (dependent) variable on its values during a previous period, using the Akaike information criterion as an estimator of the in-sample prediction error [59]. Normalized units of low-frequency and high-frequency power were calculated as the low- and high-frequency power divided by the difference (total power – very-low-frequency power) × 100 [60, 61]. Total power, the major component in the divisor used for computing normalized HRV units as applied in the study, increases with sympathetic activation, whereas vagal activation produces the opposite effect [62]. Efferent vagal activity is the major contributor to the high-frequency HRV component, as evidenced by clinical and experimental interventions, such as electrical vagal stimulation, muscarinic receptor blockade, and vagotomy [63]. More controversial is the interpretation of the low-frequency component, which is commonly interpreted as a marker of sympathetic modulation [59, 64]. Alternatively, experts consider the low-frequency HRV component as a measure reflecting the balance between the sympathetic and parasympathetic autonomous nervous outflow [59, 64]. A more recently proposed interpretation of low-frequency HRV is that it does not primarily reflect sympathetic efferent nervous outflow to the heart but rather baroreflex function [65]. Consequently, according to some experts, manipulations, and drugs that change low-frequency power or the low-to-high-frequency ratio do not affect autonomic nervous outflow to the heart in a direct manner but modulate these outflows via baroreflex arches [65].

**Fig. 2:**
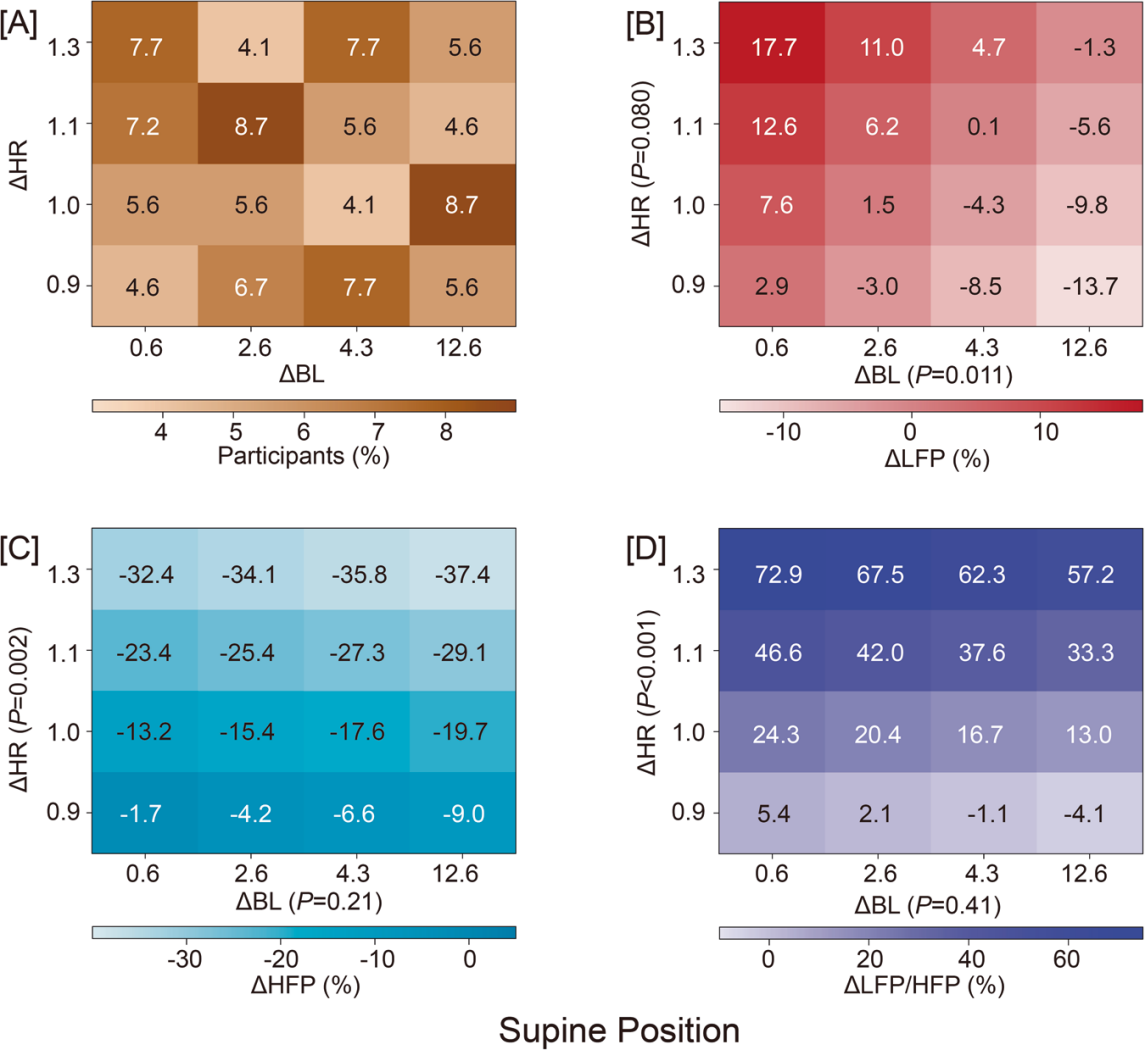
Heatmaps relating the percent changes in the heart rate variability indices with the baseline-to-follow-up heart rate ratio (ΔHR) and the follow-up-to-baseline blood lead concentration ratio (ΔBL) in the supine position. Panel **A** shows the percent of workers (*N* = 195) in each cell in the cross-classification between the baseline-to-follow-up heart rate ratio (vertical axis) and the follow-up-to-baseline blood lead concentration ratio (horizontal axis). ΔLFP, ΔHLP, and ΔLFP/HFP indicate the percent changes in low-frequency power (**B**), high-frequency power (**C**), and the low-to-high-frequency ratio (**D**). Mixed models accounted for sex, the baseline value and the change during follow-up in age, mean arterial pressure, serum insulin, hemoglobin, room temperature during the examination, season, and observer (random effect). Reproduced from ref. [46], which was published was an open-access article under the terms of the Creative Commons Attribution Non-Commercial-NoDerivs License

The analyses of heart rate and HRV [46] included 195 workers (91.3% men; mean age, 27.8 years). Study participants with irregular heart rhythm or taking antihypertensive agents or neuroactive drugs (benzodiazepines, neuroleptics, antiepileptics, sympathomimetics, amphetamines, and recreational drugs) were excluded from the analyses related to heart rate and HRV. In analyses stratified by quartiles of blood lead changes, trends in heart rate and HRV derived by the Fourier or autoregressive approach did not reveal a dose‒response curve [46]. In multivariable-adjusted mixed models, heart rate and HRV were unrelated to blood lead. The expected associations between HRV and heart rate changes were preserved, with no differences between baseline and follow-up. Along similar lines, the orthostatic heart rate responses were not altered by increasing lead exposure. Thus, a greater than 3-fold increase in blood lead did not affect autonomous neural function (Fig. 2), as captured by HRV [66].

### Peripheral nerve conduction velocity

Peripheral nerve conduction velocity (NCV) is a common test of neurological function in lead-exposed workers [67, 68]. The study nurses used a handheld device and related software (Brevio Nerve Conduction Monitoring System, NeuMed, West Trenton, NJ) to stimulate the left and right median nerve at a gradually increasing voltage until the maximum compound motor action potential of the short thumb abductor muscle was reached [66].

Peripheral NCV was assessed in 192 workers. From the lowest to the top quartile of the distribution of the follow-up-to-baseline blood lead concentration ratio, the percent changes in latency time were 2.91% (CI, −0.89 to 6.85%; *p* = 0.14), 0.86% (CI, −2.48 to 4.32%; *p* = 0.064), 2.14% (CI, −0.86 to 5.23%; *p* = 0.057), and 2.57% (CI, −0.93 to 6.19%; *p* = 0.67); the *p* value for linear trend was 0.98. The percent changes in latency time associated with a doubling of blood lead from baseline to follow-up were −0.05% (CI, −0.57 to 0.46%; *p* = 0.84) unadjusted and ‑0.09% (CI, −0.58 to 0.40%; *p* = 0.72) fully adjusted for confounders.

### Neurocognitive function

The literature relating neurocognitive function to lead exposure in studies of the general population [69–75] or workers [76–79] with a cross-sectional [69–71, 73, 74, 76], case‒control [77, 79] or longitudinal design [72, 75, 78] is contradictory. Similarly, two systematic reviews [20, 80], including 22 studies of exposed and unexposed workers but using different statistical methods, concluded that there was an inverse [80] or a null [20] association between neurocognition and occupational lead exposure. Unexposed and exposed blood lead levels in workers were unavailable in over 10 studies [20]. None of the studies compared blood lead levels before and after exposure [20]. None of the individual studies was conclusive. Lack of true measures of preoccupational exposure was a major issue that obscured the true relation between neurocognitive function and lead exposure for blood lead levels below 70 µg/dl [20].

**Fig. 3:**
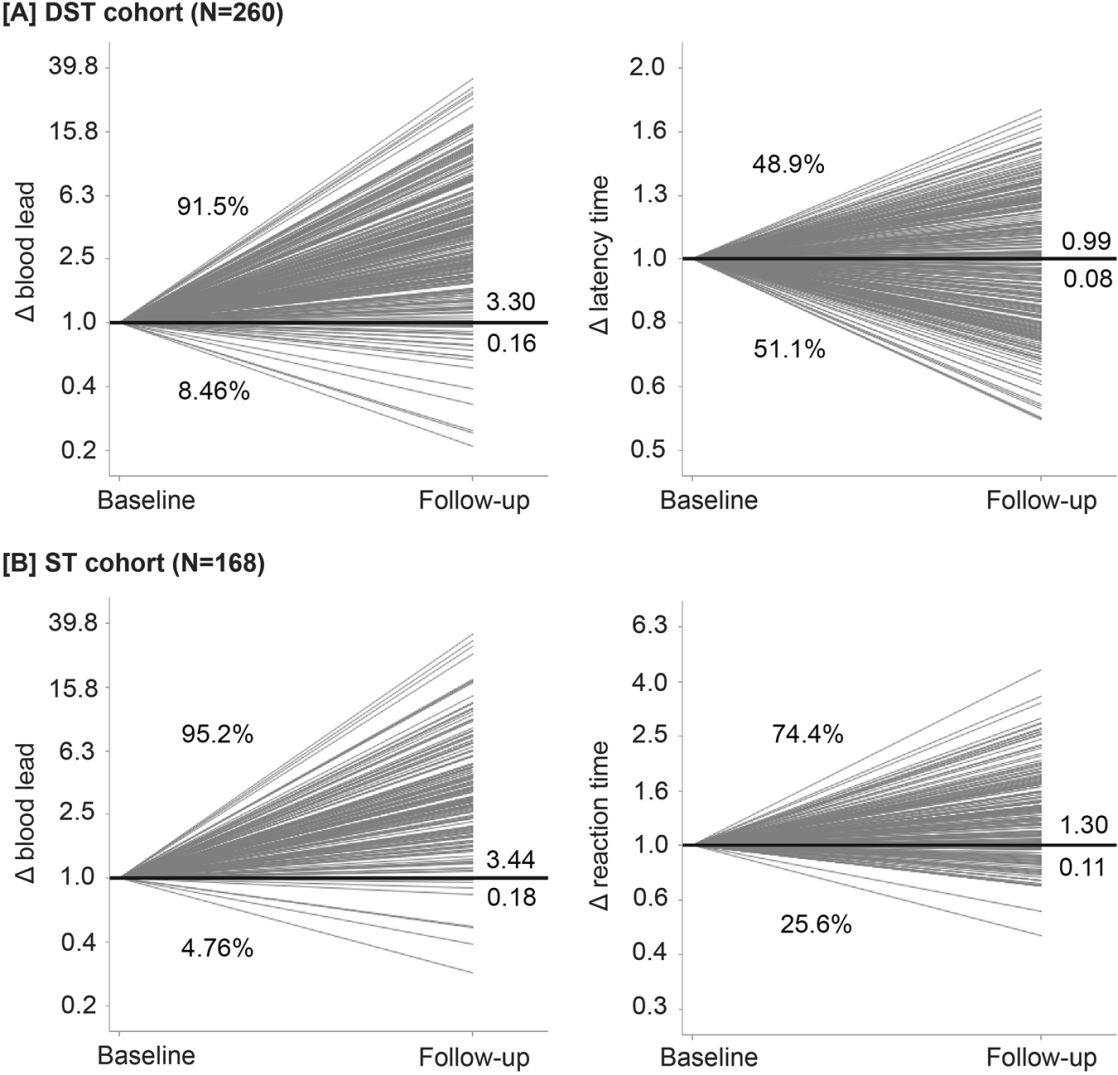
Baseline-to-last-follow-up ratios (Δ) in blood lead (**A**, **B**), latency time in the digit-symbol test in the DST cohort (**A**), and mean reaction time in the incongruent trials in the ST cohort (**B**). [DST digit-symbol test, ST Stroop test]. The numbers on the right side of the line graphs represent the mean ratio (above the unity line) and its SE (below the unity line). Percentage values represent the number of workers with a ratio greater than or less than unity. Reproduced from ref. [45], which was published was an open-access article under the terms of the Creative Commons Attribution Non-Commercial-NoDerivs License

SPHERL addressed this knowledge gap [45]. The neurocognitive tests included the computerized version of the digit-symbol test and the Stroop test, as published by Xavier Educational Software Ltd, Bangor, Wales, UK, using a laptop with touch screen [45]. The digit-symbol test measures processing speed, working memory, visuospatial processing, and attention [81]. The Stroop test was used to measure the impact of lead exposure on the Stroop effect, which is related to selective attention. The digit-symbol test was administered to 260 participants (11.9% women; 46.9%/45.0% whites/Hispanics; mean age 29.4 years), and the Stroop test was administered to 168 [45]. In unadjusted analyses (Fig. 3) and in fully adjusted models, none of the associations of the changes in the digit-symbol and Stroop test results with the blood lead changes reached statistical significance (*p* ≥ 0.12) [45].

## Perspectives

Lead exposure represents an environmental risk that should be addressed worldwide. To re-evaluate the health risks possibly associated with present-day lead exposure, a two-pronged approach was applied, first assessing recently published population metrics [25–27] and then summarizing the SPHERL results [45, 47, 48, 66]. Considering health preservation at the population level, health metrics might gain credibility by addressing the following issues: (i) ensuring the use of health data (e.g., blood pressure) in relation to present-day lead exposure levels; (ii) retesting the presumed pathogenic pathway leading from hypertension to both fatal and nonfatal adverse health outcomes; (iii) narrowing the range of cardiovascular complications potentially associated with lead exposure; (iv) developing risk models accounting for multimodal exposure to risk factors and pollutants, thereby reducing residual confounding; and (v) setting no-risk thresholds at blood lead levels that are not lower than what is achievable given the naturally occurring background sources of lead exposure. SPHERL was an ethically performed real-world experiment. The major strength of this cohort study was that it accounted for interindividual variability in the responses to a threefold blood lead increase with full documentation of the baseline values in the biomarkers of effect and exposure. Additionally, although residual confounding by unmeasured risk factors can never be excluded in observational studies, SPHERL did address a wide array of potential confounders. Nevertheless, SPHERL has limitations to be addressed in future research. First, the attrition rate among the workers who participated in the baseline examination but defaulted from follow-up amounted to over 40%, mainly because they left employment. According to the published SPHERL protocol [19], the anticipated attrition rate was 50%. To meet the sample size required to address hypertension and renal dysfunction as the primary endpoint, 500 workers had to be enrolled. Second, the small sample size and the limited 2-year follow-up of the current SPHERL cohort warrant a cautious interpretation of the findings. Third, the healthy worker effect [82] might partially account for the nonsignificant results in relation to lead exposure in this occupational cohort with a mean age of 29.7 years. The current observations should not be unthoughtfully generalized and might therefore not be applicable to older individuals or patients with comorbidities, such as diabetes [83], which increases the vulnerability of renal function. Most studies summarized in this review were conducted in high-income countries. In middle- and low-income countries, environmental regulations are less stringent or are only loosely applied. For instance, in Pakistan, blood lead was significantly higher in fume-exposed workers (median 61.2 µg/dl, range 21.2–171.1 µg/dl) than in controls (median 23.8 µg/dl, range 10.2–44.1 µg/dl) [84]. A recent cross-sectional population survey in Haiti (*n* = 2504) reported a geometric mean blood lead level of 4.73 µg/dl; of these participants, 42.3% had a blood lead level of 5 µg/dl or higher [85]. Finally, coexposure to other metals, such as cadmium, is common in lead recycling plants. This metal accumulates in the kidneys with a half-life exceeding 30 years [86]. Cadmium is an established renal toxicant that adversely affects renal tubular and glomerular function [87].

## Supplementary information

SUPPLEMENTARY MATERIALS
