# Supplementary material for: Public and occupational health risks related to lead exposure updated according to present-day blood lead levels
Source: Hypertens Res. 2022 Oct 18;46(2):395–407. doi: 10.1038/s41440-022-01069-x (PMC9899691; doi:10.1038/s41440-022-01069-x)
Supplement: Supplementary file 1 — SUPPLEMENTARY MATERIALS [file 41440_2022_1069_MOESM1_ESM.doc]

**HYPERTENSION RESEARCH**

**SUPPLEMENTARY MATERIALS**

***Public and occupational health risks related to lead exposure updated according to contemporary blood lead levels***

*Yu-Ling Yu, Wen-Yi Yang, Azusa Hara, Key Asayama, Harry A. Roels, Tim S. Nawrot, Jan A. Staessen*

**Table of contents**

**page**

**Supplementary Table 1** Chronic Kidney Disease Epidemiology Collaboration equations for estimating
glomerular filtration rates derived from serum creatinine, serum cystatin C
and both 2

**Supplementary Figure 1** Distributions of blood lead at baseline and last follow-up and of the
last-follow-up-to-baseline blood lead ratio 3

**Supplementary Figure 2** Heat maps relating the changes in glomerular filtration to their baseline values
and the baseline blood lead concentration 4

**Supplementary Figure 3** Boxplots showing the distributions of the glomerular filtration rate derived from
serum creatinine, serum cystatin C, or both serum creatinine and cystatin C
by study phase and work shift 5

**Supplementary Table 1**

Chronic Kidney Disease Epidemiology Collaboration equations for estimating glomerular filtration rates derived from serum creatinine, serum cystatin C or both

| Variable | Sex | Serum creatinine (mg/dl) | Serum cystatin C (mg/l) | Equation for estimating GFR |
| --- | --- | --- | --- | --- |
| eGFRcrt | Female | ≤ 0.7 |  | 144 × (Scr / 0.7)-0.329 × 0.993Age [× 1.159 if black] |
|  |  | > 0.7 |  | 144 × (Scr / 0.7)-1.209 × 0.993Age [× 1.159 if black] |
|  | Male | ≤ 0.9 |  | 144 × (Scr / 0.7)-0.411 × 0.993Age [× 1.159 if black] |
|  |  | > 0.9 |  | 144 × (Scr / 0.7)-1.209 × 0.993Age [× 1.159 if black] |
| eGFRcys | Female or male |  | ≤ 0.8 | 133 × (Scys / 0.8)-0.499 × 0.996Age [× 0.932 if female] |
|  |  |  | > 0.8 | 133 × (Scys / 0.8)-1.328 × 0.996Age [× 0.932 if female] |
| eGFRcc | Female | ≤ 0.7 | ≤ 0.8 | 130 × (Scr / 0.7)-0.248 × (Scys / 0.8)-0.375 × 0.995Age [× 1.08 if black] |
|  |  |  | > 0.8 | 130 × (Scr / 0.7)-0.248 × (Scys / 0.8)-0.711 × 0.995Age [× 1.08 if black] |
|  |  | > 0.7 | ≤ 0.8 | 130 × (Scr / 0.7)-0.601 × (Scys / 0.8)-0.375 × 0.995Age [× 1.08 if black] |
|  |  |  | > 0.8 | 130 × (Scr / 0.7)-0.601 × (Scys / 0.8)-0.711 × 0.995Age [× 1.08 if black] |
|  | Male | ≤ 0.9 | ≤ 0.8 | 135 × (Scr / 0.7)-0.207 × (Scys / 0.8)-0.375 × 0.995Age [× 1.08 if black] |
|  |  |  | > 0.8 | 135 × (Scr / 0.7)-0.207 × (Scys / 0.8)-0.711 × 0.995Age [× 1.08 if black] |
|  |  | > 0.9 | ≤ 0.8 | 135 × (Scr / 0.7)-0.601 × (Scys / 0.8)-0.375 × 0.995Age [× 1.08 if black] |
|  |  |  | > 0.8 | 135 × (Scr / 0.7)-0.601 × (Scys / 0.8)-0.711 × 0.995Age [× 1.08 if black] |

eGFRcrt, eGFRcys, and eGFRcc refer to the glomerular filtration rate estimated from serum creatinine, serum cystatin C or both. To convert serum creatinine from mg/dl to µmol/l, multiply by 88.42; to convert cystatin C from mg/l to nmol/l, multiply by 74.9. Reproduced with permission from
*N Engl J Med.* 2012;367:20-29 (reference 52).


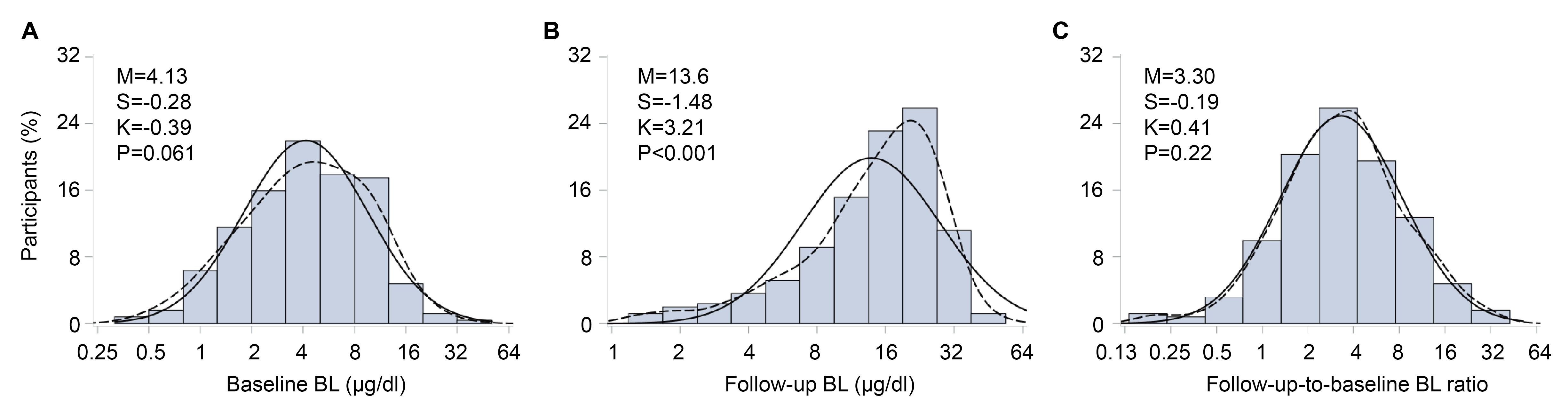


**
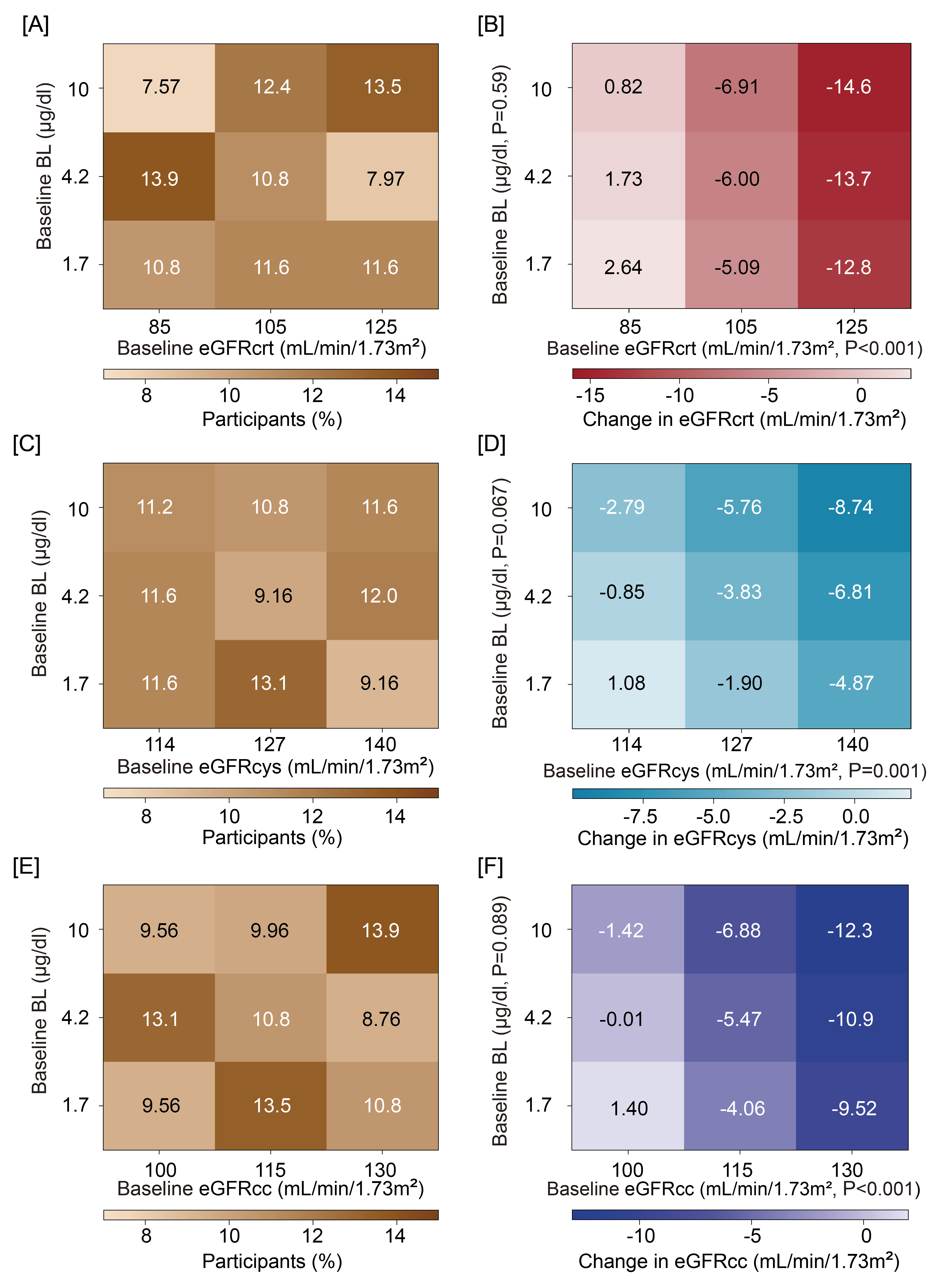
**

**Supplementary Figure 1**

Distributions of blood lead at baseline (A) and last follow-up (B) and of the last-follow-up-to-baseline blood lead ratio (C). The solid and dotted lines represent the normal and kernel density distributions. *P* values are for departure of the actually observed distribution from normality according to the Shapiro-Wilk statistic. M indicates the geometric mean. Skewness (S) and kurtosis (K) were computed as the third and fourth moments about the mean divided by the cube of the standard deviation. Reproduced with permission from reference 48.

**Supplementary Figure 2**

Heat maps relating the changes in glomerular filtration rate to their baseline values and the baseline blood lead (BL) concentration. Participants were cross-classified by thirds of the distributions of the baseline BL and the baseline glomerular filtration rates, as derived from serum creatinine (eGFRcrt; panels A and B), serum cystatin C (eGFRcys; panels C and D) or both (eGFRcc; panels E and F). The percentage of participants contributing to run of the heat maps is given in panels A, C, and E. Association sizes are point estimates corresponding with the thick marks and were derived from fully adjusted mixed models, which accounted for sex, age, follow-up duration, the time of day of blood sampling (daytime *vs* nighttime), body mass index, change in body weight, the follow-up-to-baseline BL ratio, and the baseline values of and changes during follow-up in smoking status, mean arterial pressure, antihypertensive medication (yes *vs* no), total-to-HDL cholesterol ratio, and γ‑glutamyltransferase. The *P*‑values of the interaction terms between baseline BL and the baseline eGFR were 0.50, 0.11 and 0.70 for eGFRcrt (B), eGFRcys (E) and eGFRcc (F), respectively. Reproduced with permission from reference 48.


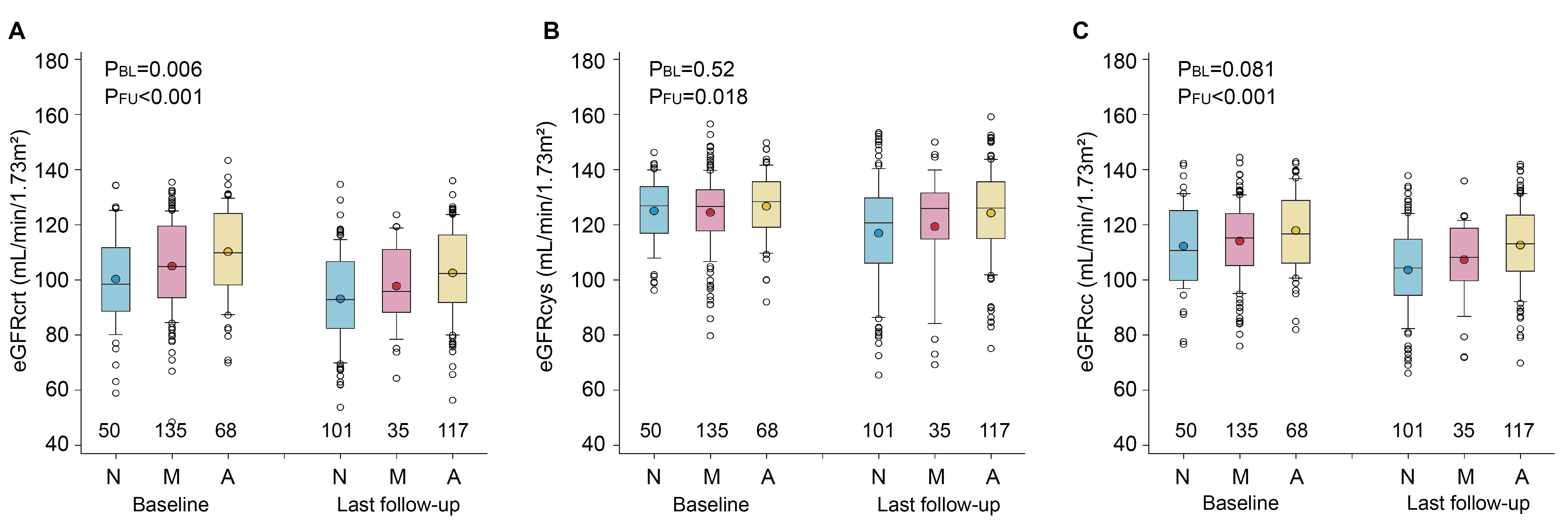


**Supplementary Figure 3**

Boxplots showing the distributions of the glomerular filtration rate derived from serum creatinine (eGFRcrt; panel A), serum cystatin C (eGFRcys; panel B), or both serum creatinine and cystatin C (eGFRcc; panel C) by study phase (baseline and last follow-up) and by work shift (night [N], morning [M], and afternoon [A]). The central line, the upper and lower lines, and the upper and lower caps represent the median, interquartile range, and the 10th to 90th percentile interval. The arithmetic means and extreme measurements are represented by circles inside the box and outside the whiskers, respectively. The number of data points contributing to each whisker plot is given along the horizontal axis. *P* values denote the significance of the overall difference between the estimates of the glomerular filtration rate by time of day for baseline (*PBL*) and follow-up (*PFU*). Reproduced with permission from reference 48.
